# Coincidence of HPV11-Positive Urethral Condyloma Acuminatum and HPV-Negative Multiple Bladder Papillomas in a Female

**DOI:** 10.1155/2012/602819

**Published:** 2012-03-04

**Authors:** Natsuko Nakazaki, Masayoshi Zaitsu, Koji Mikami, Shunsuke Yui, Ayumi Kanatani, Takushi Nakatani, Akiko Ito, Yuta Takeshima, Akiko Tonooka, Hideaki Oka, Tomoko Miki, Takumi Takeuchi

**Affiliations:** ^1^Department of Urology, Kanto Rosai Hospital, 1-1 Kizukisumiyoshi-cho, Nakahara-ku, Kawasaki 211-8510, Japan; ^2^Department of Pathology, Kanto Rosai Hospital, 1-1 Kizukisumiyoshi-cho, Nakahara-ku, Kawasaki 211-8510, Japan; ^3^Department of Infectious Diseases, Kanto Rosai Hospital, 1-1 Kizukisumiyoshi-cho, Nakahara-ku, Kawasaki 211-8510, Japan

## Abstract

Human papillomaviruses (HPVs) are associated with proliferative lesions in a variety of human epithelial types. A 38-year-old female presented with a diagnosis of urethral condyloma acuminatum. She underwent transurethral resection of the urethral condyloma. At that time, multiple (five) bladder tumors were simultaneously found and also removed by transurethral resection. Four of the bladder tumors were diagnosed as squamous papilloma, and the other was urothelial inverted papilloma. Postoperative course was uneventful. Genomic DNA was extracted from 10 *μ*m thick sections of each bladder tumor as well as urethral condyloma. Then, 16 types of HPV DNA sequences were assessed with the PapiPlex method using genomic DNA samples extracted from each bladder tumor as well as urethral condyloma. HPV-11 was detected in DNA extracted from the urethral condyloma, while no HPV DNA sequences were positive in any of the genomic DNA samples extracted from the bladder tumors.

## 1. Introduction

 Human papillomaviruses (HPVs) are associated with benign and malignant proliferative lesions in a variety of human epithelial types ranging from papilloma, verrucae, condyloma acuminatum, epithelial hyperplasia, dysplasia, to carcinoma [1–3]. With the introduction of the sensitive polymerase chain reaction (PCR) technique in mid-1980s, HPVs in various tissues have been vigorously investigated. There is close association between types of infected HPVs and subsequent diseases. For example, HPV-6 and 11 (low-risk HPVs) cause up to 90* *% of condyloma acuminata, while HPV-16 and 18 (high-risk HPVs) induce up to 70* *% of cervical neoplasias [4]. Association between HPV infection and urinary tract tumors is still under investigation. Here we show a case of urethral condyloma acuminatum and concomitant multiple bladder papillomas, and existence of HPVs in each of the tumors was assessed by the PCR.

## 2. Case Presentation

**Figure 1:**
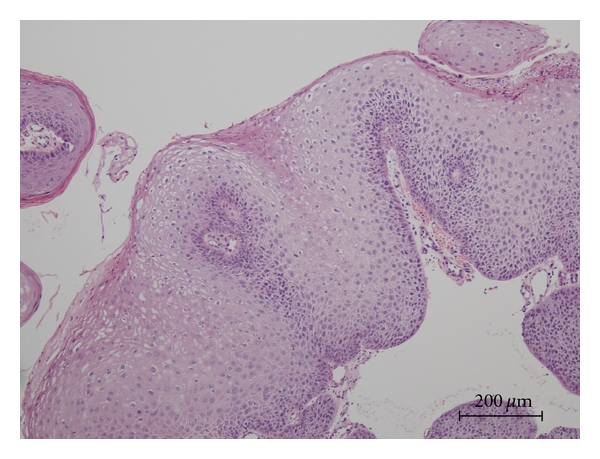
Hematoxylin and eosin staining of HPV11-positive urethral condyloma acuminatum showing koilocytosis and parakeratosis.

**Figure 2:**
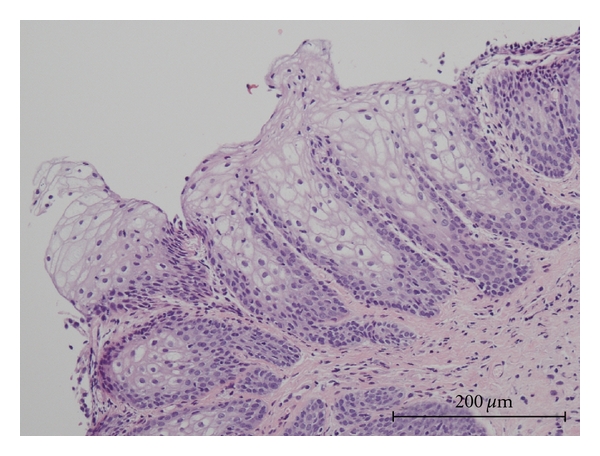
Hematoxylin and eosin staining of bladder squamous papilloma (HPV negative).

**Figure 3:**
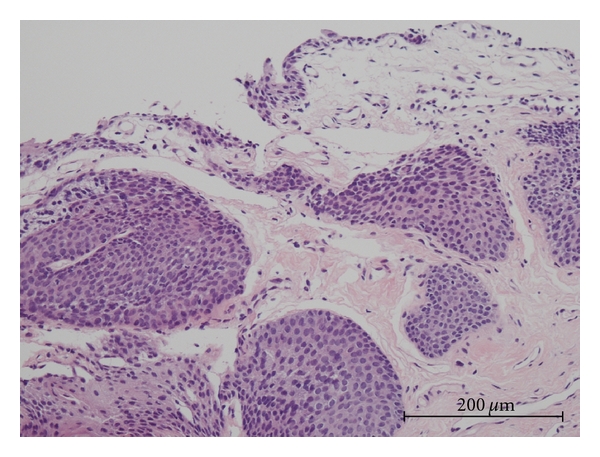
Hematoxylin and eosin staining of bladder urothelial inverted papilloma (HPV negative).

A 38-year-old female presented with a diagnosis of urethral condyloma acuminatum. There were no condylomatous lesions in the gynecological area. She underwent transurethral resection of the urethral condyloma. At that time, multiple (five) bladder tumors of less than 1 cm in diameter were simultaneously found and also removed by transurethral resection. Tissues were fixed in 10% formalin, then embedded in paraffin. Sections were stained with hematoxylin and eosin (HE). Four of the bladder tumors were diagnosed as squamous papilloma (Figure 2), and the other was urothelial inverted papilloma (Figure 3) by HE staining. Histology of the urethral lesions showed koilocytosis and parakeratosis supporting the diagnosis as condyloma acuminatum (Figure 1). Post-operative course was uneventful.

**Figure 4:**
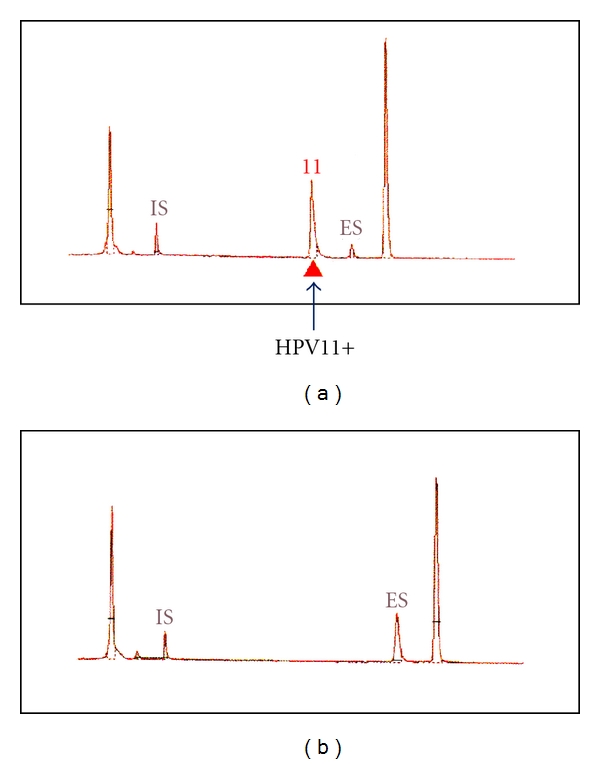
Detection of HPV sequences by the PapiPlex method. Upper panel: urethral condyloma showing HPV11 sequences (arrow), lower panel: a squamous papilloma with no HPV sequences.

Genomic DNA was extracted from 10 *μ*m thick sections of each bladder tumor as well as urethral condyloma. Then, HPV-6, 11, 16, 18, 30, 31, 33, 35, 39, 45, 51, 52, 56, 58, 59, and 66 DNA sequences were assessed at one time with the PapiPlex method [5] using those DNA samples as templates. HPV-11 DNA sequences were detected in DNA extracted from the urethral condyloma, while no HPV DNA sequences were positive in any of the genomic DNA samples extracted from the bladder tumors (Figure 4).

## 3. Discussion

In the urinary tract, linkage of urothelial cancer and HPV infection is still controversial. Saltzstein et al. did not detect HPV-6, 11, 16, 18, 31, and 33 in any of 33 urothelial cancer cases regardless of their invasiveness [6], while Chan et al. detected HPV-18 in 6 out of 20 papillary urothelial cancers [7]. In addition, Roussel et al. detected HPV in one out of 6 non-invasive urothelial cancers [8], and Mincione et al. detected HPV-31, 33, and 51 in one of 18 urothelial cancers [9]. Bladder cancer caused by infection of *Schistosoma haematobium* may be unique. HPV-16 was detected in all of 27 bladder cancers due to schistosomiasis [10]. For other bladder neoplasias than carcinomas, HPV-16 or 18 DNA sequences were reported to be detectable in 87.5% [11] and 20% [7] of urothelial inverted papillomas. Squamous papillomas and verrucous carcinomas of the bladder were HPV negative, while condyloma acuminatum was HPV positive [12].

In the present case, HPV-11 was positive in urethral condyloma acuminatum, but none of the HPVs was detected in urothelial and squamous papillomas in the bladder. Those papillomas in the patient may not be caused by HPV infection and were concomitant with urethral condyloma by chance, although the multiplicity of the simultaneous papillomas elicits the possibility of the same genesis. Similarly to this case, Olsen et al. detected HPV-6 and 11 in a male patient with urethral condyloma, while no HPVs were demonstrable in recurring papillomatous urothelial cancers in the same patient [13]. When papillomas in the urinary tract concomitant with genitourinary condyloma acuminatum are resected, HPV DNA sequences in the papillomas as well as those in the condyloma need to be assessed. If HPV types are the same in the papillomas and the condyloma, both lesions are supposed to be caused by the same HPV(s) and the risk of recurrence may be higher requiring closer follow-up. In conclusion, HPV-11 was positive in urethral condyloma acuminatum in a female patient, but all HPV sequences were negative in concominant multiple bladder squamous papillomas and urothelial inverted papilloma.
